# Phytochemical Investigation of an *Ostrya carpinifolia* L. Extract: An Effective Anti‐Pollution Cosmetic Active Ingredient.

**DOI:** 10.1002/cbdv.202402139

**Published:** 2024-11-07

**Authors:** Manon Trinel, Camille Dubois, Pauline Burger, Hortense Plainfossé, Stéphane Azoulay, Gregory Verger‐Dubois, Xavier Fernandez

**Affiliations:** ^1^ Université Côte d'Azur CNRS ICN Parc Valrose, CEDEX 2 06108 Nice France; ^2^ NissActive Pépinière Innovagrasse Espace Jacques-Louis Lions 4 traverse Dupont 06130 Grasse France

**Keywords:** *Ostrya carpinifolia* L., Anti-pollution activity, Cosmetic bioassays, Flavonoids, Phenolic compounds

## Abstract

*Ostrya carpinifolia* L., a member of the Betulaceae family, is a tree endemic to the Mediterranean basin that is well known for the hardness of its wood. In this study, we assess the anti‐pollution activities of a hydroalcoholic extract of *O. carpinifolia* twigs using several judiciously selected *in vitro* cosmetic bioassays. The extract's capacity to counteract excessive production of reactive oxygen species following a cutaneous exposure to atmospheric pollution was evaluated using a combination of several antioxidant assays: DPPH, FRAP and β‐carotene bleaching assays. These antioxidant assays were complemented by anti‐elastase, anti‐collagenase, anti‐hyaluronidase and anti‐lipoxygenase assays to evaluate the capacity of the extract to preserve the integrity of the skin. The hydroalcoholic extract of *O. carpinifolia* demonstrates intriguing biological antioxidant activities, with approximately 50 % inhibition observed in DPPH and β‐carotene assays. Furthermore, its anti‐lipoxygenase, anti‐hyaluronidase, and anti‐collagenase activities are noteworthy, exceeding 50 % inhibition. The two major compounds of *O. carpinifolia* ethanolic extract were isolated and identified as myricitrin **(1)** and quercitrin **(2)**. Myricitrin and quercitrin exhibit antioxidant and anti‐hyaluronidase properties; we explored the correlation of these properties with the activity of the crude hydroalcoholic extract. Notably, these compounds have not been previously described in the Ostrya genus.

## Graphical abstract

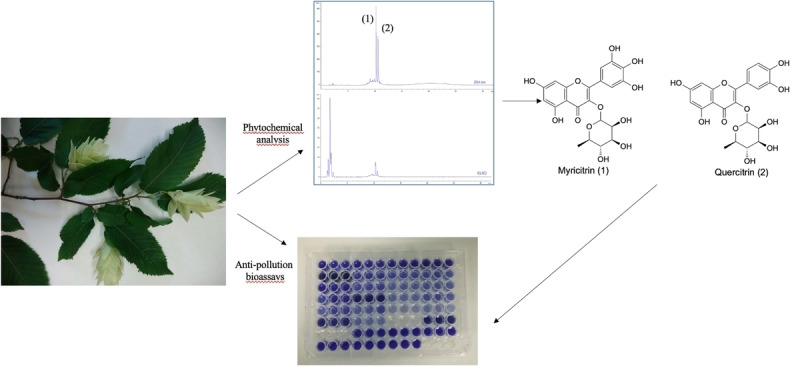

## Introduction

The cosmetic industry is an industrial sector that consistently seeks out new natural products to develop new ingredients and active components. This search is driven by the growing consumer preference for natural options, which has been particularly evident over the past several years due to an increased ecological awareness. Consumers are actively seeking cosmetics that they perceive as more environmentally friendly, often referred to as “greener” cosmetics. They believe these options are not only healthier and safer for themselves but also for environment.[^1^, ^2^, ^3^] However, they are also more concerned about the real efficacy of the products they are using daily and are paying close attention to the properties attributed to products. Cosmetic manufacturers must therefore now provide evidence of the activities claimed on their products’ packaging through specific, scientifically approved bioactivity assays and safety information.^[4]^

The cosmetics industry has witnessed the emergence of specific trends over time, one notable example being the anti‐pollution trend, which appeared in Asia about ten years ago and has now spread throughout the world.^[5]^ Pollution sources are quite diverse and daily exposure to pollutants is increasing, especially in cities. Among the principal pollutants are Particulate Matter (PM), ozone, carbon monoxide, Polycyclic Aromatic Hydrocarbons (PAHs), Trace Metal Elements (TMEs), cigarette smoke, blue light, and other agents.[^6^, ^7^] They alter the skin integrity in many different ways, and their individual effects may vary in intensity depending on the bodily area exposed.[^7^, ^8^, ^9^, ^10^]

Despite this variety, several common harmful outcomes resulting from exposure to pollutants are worth highlighting. One of the principal effects is the increased production of Reactive Oxygen Species (ROS) inducing harmful oxidative stress. ROS are highly reactive and toxic compounds present in cells, particularly in the mitochondria. Their levels are typically regulated by antioxidant molecules like vitamin E and enzymes such as superoxide dismutase.[^11^, ^12^] Oxidative stress arises when the quantity of pro‐oxidant molecules outweighs that of antioxidants. This affects proteins, lipids, sugars, DNA and therefore the cells and their membranes. At the cutaneous level, this production of ROS leads to the disruption of the skin's natural balance and mechanisms.^[6]^ Prolonged or repeated exposure to these pro‐oxidants disrupts the skin's redox balance, causing various alterations: premature aging (dull complexion, dry skin, wrinkles, pigmentation spots), changes in sebum secretion quality and quantity, inflammation, and allergic conditions (atopic dermatitis, eczema, psoriasis, acne, etc.). Thus, antioxidant activity is crucial in neutralizing ROS and preventing these adverse skin effects, making it an essential mechanism in the anti‐pollution efficacy of cosmetic applications.^[8]^

Pollutants are also responsible for inducing an inflammatory cascade that results in a weakening of the skin's defenses, i. e., a loss of the shield function.^[13]^ Finally, the last major phenomenon induced by pollution is the destruction of the cutaneous microflora to a greater or lesser extent, which makes way for pathogenic bacteria.^[13]^ Because of these numerous and diverse phenomena, no agreed anti‐pollution index, as for solar products for example, is established yet. Some bioassays are used now to demonstrate the anti‐pollution activity of a cosmetic active but most of them remain too expensive to be extensively used to screen the activities of a large selection of ingredients because of the use of specific pollution exposure system or because they are carried out on skin cells or volunteers. Also, such assays usually only target a single effect of the cutaneous exposure to atmospheric pollution.^[14]^

The following table (**Table** 1) summarizes the key atmospheric pollutants, their detrimental effects on skin, and the corresponding assays used in this study to evaluate the protective effects of the extract. These assays were chosen to simulate real‐world skin exposure to pollutants and assess the extract's efficacy in mitigating oxidative stress, inflammation, and degradation of essential skin components.

**Table 1:** Relation between skin exposure to pollutants and the selected assays.

Pollutant	Effect on skin	Corresponding assay
Fine particles (PM), Ozone (O3), NOx, SOx, CO, Lead	Oxidation of skin cells, formation of reactive oxygen species (ROS), oxidative stress, premature skin aging (wrinkles, dull complexion, dry skin)	DPPH, FRAP, Beta‐carotene: Antioxidant tests to measure the capacity to neutralize ROS and restore skin's redox balance.
All pollutants	Skin inflammation, barrier function deficiency, allergic conditions (dermatitis, eczema, psoriasis)	Lipoxygenase: Measures the inflammatory reaction induced by pollutant exposure.
Ozone, PAHs, VOCs	Degradation of hyaluronic acid, leading to loss of skin hydration and sagging	Hyaluronidase: Measures the degradation of hyaluronic acid, which affects skin hydration.
PM, Ozone, NOx, SOx, VOCs	Degradation of collagen and elastin fibers, leading to loss of skin firmness and elasticity	Elastase and Collagenase: Enzymatic assays to evaluate the degradation of collagen and elastin fibers, responsible for skin firmness and tension.

This paper describes the selection of *in vitro* anti‐pollution assays and the bio‐guided fractionation process of a hydroalcoholic 50/50 (EtOH/H2O, v/v) extract obtained from *Ostrya carpinifolia* twigs. This process resulted in the isolation and identification of two active compounds, which were recognized but have not previously described within the genus *Ostrya*. A series of bioassays were conducted to assess the anti‐pollution activities of the crude extract, as well as the enriched fractions and individual pure compounds derived from it. Finally, we discuss the development of a liquid cosmetic active ingredient from *O. carpinifolia*.

## Results and Discussion

### *In vitro* Anti‐Pollution Assay Selection

**Figure 1:**
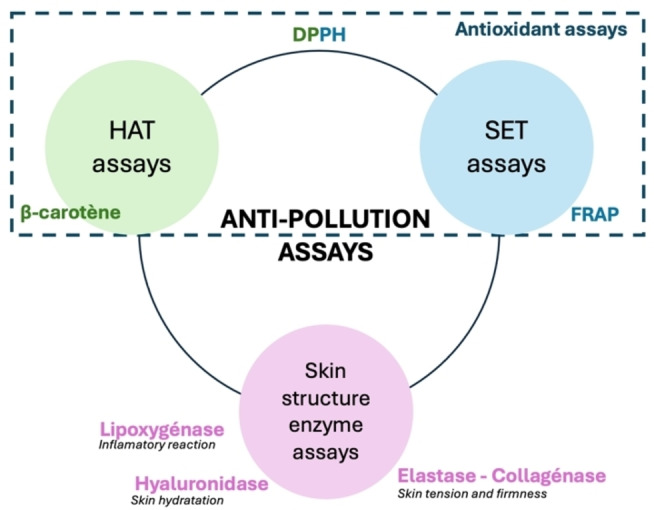
*In vitro* anti‐pollution assays used in the study.

The strategy adopted in the present article consists of testing a set of bioassays aimed at being as representative as possible of potential anti‐pollution activity against the main phenomena induced by pollutants described above. In our opinion, this set could be selected from among the already existing enzymatic assays, that are well established, repeatable, implemented by all, and well known in the scientific world. To begin with, as noted, pollution induces oxidative stress in the skin cells. Therefore, assays evaluating the antioxidant activity of natural extracts logically should be part of the anti‐pollution assays set (**Figure** 1).

Concerning the *in vitro* antioxidant assays, there are commonly three groups: Hydrogen Atom Transfer (HAT) assays,^[15]^ Single Electron Transfer (SET) assays^[16]^ and Mixed HAT and SET assays.^[17]^ HAT assays measure the capacity of antioxidant molecules to donate hydrogen to stabilize free radicals, while SET assays measure the ability to provide one or more electrons for stabilization. Mixed HAT and SET assays stabilize free radicals through both hydrogen and electron transfers, making them more generally indicative of antioxidant activity but with less specificity than the two other groups.

The DPPH assay based on the scavenging activity of the stable 1,1‐diphenyl‐2‐picrylhydrazyl radical, is the most widely used *in vitro* antioxidant assay. Therefore, we included it in the set, even though it is not considered as specific because it is a Mixed HAT and SET assay.^[17]^ It therefore seemed appropriate to supplement the DPPH assay with two other specific antioxidant assays to complete the set. We chose the Ferric Reducing Antioxidant Power (FRAP) assay^[18]^ as a SET assay that measures the potential of a sample for metal complexation or inactivation. More specifically, the FRAP assay is a colorimetric assay that measures the ability of extracts to reduce iron. This involves changing the ferric form of iron to ferrous iron via the reduction of the Fe^3+^ ‐ TPTZ (iron 2,4,6‐tripyridyls‐triazine) complex.^[19]^ For the HAT assay, we chose the β‐carotene bleaching assay, which is also a colorimetric test that measures the ability of a natural extract to counteract the oxidative degradation of β‐carotene (visible by discoloration) induced by the oxidation products of linoleic acid. For this reason, the β‐carotene bleaching assay specifically allows the study of an anti‐lipidic peroxidation potential.^[17]^

The increase of ROS production modifies the balance of pro‐oxidant/antioxidant molecules and leads to damage to the skin structure. Three of the essential enzymes that play a role in skin structure and are enhanced by pollution exposure are hyaluronidases, collagenases, and elastases. Elastases and collagenases are enzymes that respectively catalyze the hydrolysis of elastin and collagen, structural fibers playing a crucial role in the maintenance of the skin's tension and firmness.^[20]^ Hyaluronidase induces the degradation of hyaluronic acids, contributing to natural cutaneous hydration due to their strong capacity to retain water. Such degradation gradually leads to a significant increase of the tissues’ permeability.^[21]^ Finally, we have reported that pollutants trigger a permanent inflammatory reaction in the skin in response to the daily attacks of pollutants enhancing some enzymes like lipoxygenase. This is a protease that catalyzes the oxidation reactions of fatty acids or other unsaturated compounds, in other words, it governs inflammatory reactions and controls cell repair. The control and maintenance of the enzymatic balance concerning these four enzymes therefore appears to be essential to the activities of an anti‐pollution ingredient.^[22]^

Thus, the above combination of seven *in vitro* bioassays makes it possible to assess quite easily the anti‐pollution potential of a natural extract by observing its capacity to counteract the phenomena that result from cutaneous exposure to atmospheric pollutants, i. e., inflammation, oxidative phenomena, and cutaneous structural disorganization.

### Natural Agents in Anti‐Pollution Applications

Although many studies have been conducted to determine the impact of pollution on the skin, the subject's complexity has led the cosmetics industry to focus on the overall impact of pollution rather than addressing the effects of individual pollutants.^[8]^ Despite the lack of a standardized test to evaluate the overall anti‐pollution activity of an ingredient, numerous actives are currently used by cosmetic professionals to combat the harmful effects of pollution. Many of these ingredients consist of blends with different mechanisms of action or targeting various pollutants. These ingredients may include actives that prevent particle penetration into the skin, strengthen the skin barrier, provide antioxidants to combat oxidative stress, prevent collagen and elastin fiber degradation, control skin pigmentation, and possess anti‐inflammatory properties to soothe skin reactions.^[14]^

As a result, products on the market are formulated to combat oxidative stress and excessive sebum secretion, delay skin aging, and strengthen the skin's barrier function.^[8]^ Some active ingredients are used for their film‐forming properties, that is, by creating a film on the skin's surface, they physically prevent pollutant particles from adhering, thus protecting the epidermis from environmental stressors. Many products also aim to remove impurities accumulated throughout the day.^[8]^

In the current cosmetics market, there are anti‐pollution gels, creams, and masks made up of various ingredients, with claimed activities by the manufacturers. Among these are Rosa Canina fruit extract (antioxidant), Vitis Vinifera fruit extract and Moringa Oleifera seed extract (anti‐pollution), Moringa Pterygosperma seed extract (anti‐pollution, detoxifying) and Chlorella Vulgaris extract (anti‐pollution, detoxifying).^[8]^

In France, pioneers like Clarins (with products such as “Anti‐Pollution Gentle Foaming Cleanser” and “Multi‐Protection UV Day Screen”) and Biotherm (notably with their “Skin Oxygen” range) have been joined in the anti‐pollution market by brands like Nuxe, Decléor, Dermalogica, and others.^[8]^

### Evaluation of the Anti‐Pollution Potential of the Hydroalcoholic Extract of *O. carpinifolia*

Our location in a Mediterranean area that is a biodiversity hotspot^[23]^ gives us access to many species that may be useful in the search for new natural cosmetic active ingredients to respond to current issues. Hence, about twenty Mediterranean plants were initially selected to assess their potential as effective natural anti‐pollution cosmetic actives. These plants were selected based on their endemicity, availability (large presence in the region, sustainable supply, etc.), originality (no existing phytochemical scientific article or patent associated with their potential use as cosmetic active) and based on preliminary evaluation of the bioactivities of an hydroalcoholic 50/50 (EtOH/H_2_O, v/v) extract using the set of assays described above. From this selection, the hydroalcoholic extract of *Ostrya carpinifolia* L. twigs stood out and was selected for further investigation to develop a new efficient anti‐pollution active.

*Ostrya carpinifolia* L. (Betulaceae) is a tree endemic to the Mediterranean basin well known for the hardness of its red wood.^[24]^ Existing literature on *O. carpinifolia* deals mainly with its geographical distribution, the wood properties or its allergenic potential.[^24^, ^25^, ^26^, ^27^] Only few articles deal with the phytochemical composition. The presence of cardiotonic glycosides, coumarins, emodins, flavonoids, leucoanthocyanins, steroids, tannins, and terpenes has been documented in ethanolic extracts of *Ostrya carpinifolia* stems, leaves, and inflorescences; however, to our knowledge, no compounds have been isolated or identified.^[28]^ Starcevic *et al*. also provided evidence of the antioxidant activity of these three extracts using the DPPH assay.^[28]^ Moreover, the presence of phenolic compounds, including several derivatives of kaempferol, apigenin, myricetin and morin was demonstrated in *O. carpinifolia* bud exudates.[^29^, ^30^]

The 50 % hydroalcoholic extract captured our interest because it showed promising results in biological activity tests and its phytochemical profile, which is rich in compounds.

**Figure 2:**
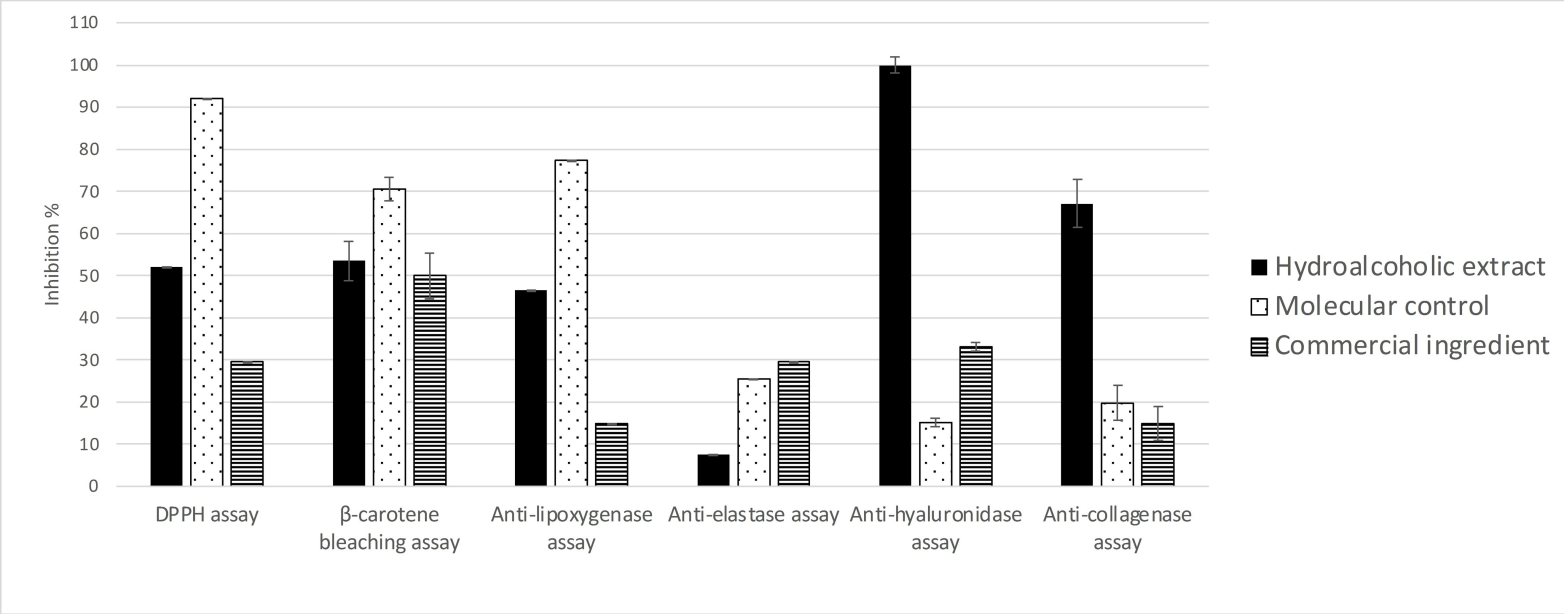
Bioactivities of the hydroalcoholic 50/50 extract of O. carpinifolia twigs compared to positive controls. All values are mean ± SEM, done in triplicate.

The *in vitro* anti‐pollution potential of the hydroalcoholic 50/50 extract of the twigs of *O. carpinifolia* was assessed through seven *in vitro* bioassays. As shown in **Figure** 2, the crude extract of *O. carpinifolia* displays promising activities, positioning it as a strong candidate for the development of an effective anti‐pollution cosmetic active. Notably, the crude extract demonstrates a strong anti‐hyaluronidase activity at 100 μg/mL (100.00±1.86 %). It also presents anti‐inflammatory and anti‐collagenase activities that are three and five times more efficient, respectively, than the corresponding commercial positive control. In addition, it shows a good β‐carotene bleaching activity in comparison with the commercial ingredient (51.00±0.01 % vs 50.00±0.01 %). The iron reducer power is about 7.85±1.09 mM/LFe(II) compared with the commercial extract which presented 0.42±0.005 mM/LFe(II) at the same concentration.

**Figure 3:**
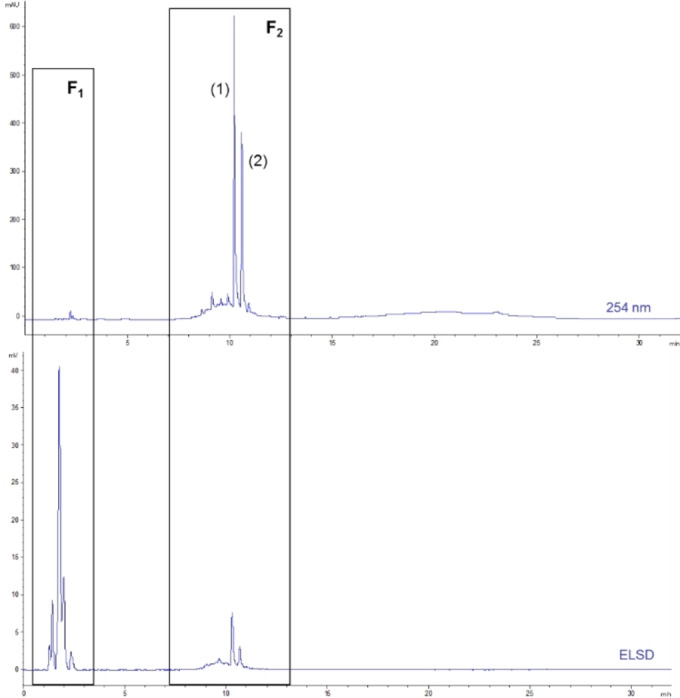
HPLC chromatograms of *O. carpinifolia* hydroalcoholic 50/50 extract at 254 nm and with ELSD obtained with gradient 1, presenting the two groups of compounds F_1_ and F_2_ and the compounds (**1**) and (**2**).

The analysis of this crude extract aimed to identify the phytochemical profiles and the families of compounds it contains. HPLC‐DAD/ELSD analysis (**Figure** 3) demonstrates that is mainly composed of two groups of compounds. The first one, named F_1_, which is eluted before 3 min, appears to consist exclusively of highly polar compounds. These compounds do not exhibit absorbance at 254 nm, suggesting sugars, amino acids, peptides, small carboxylic acids, etc. The second group (or fraction), named F_2_, eluted between 7 and 13 min, seems to be constituted by a multitude of polyphenols and flavonoids. The section labeled F_2_ in **Figure** 3 contains two major compounds, numbered (**1**) and (**2**). These two compounds dominate within the group of less polar molecules, such as polyphenols. Considering the chemical composition of this extract, it appears essential to separate these two groups of compounds to determine whether only one is responsible for the bioactivities evidenced or if these are the result of a synergy between the two groups of molecules.

### Evaluation of the Biological Activities of Fractions F1 and F2

**Figure 4:**
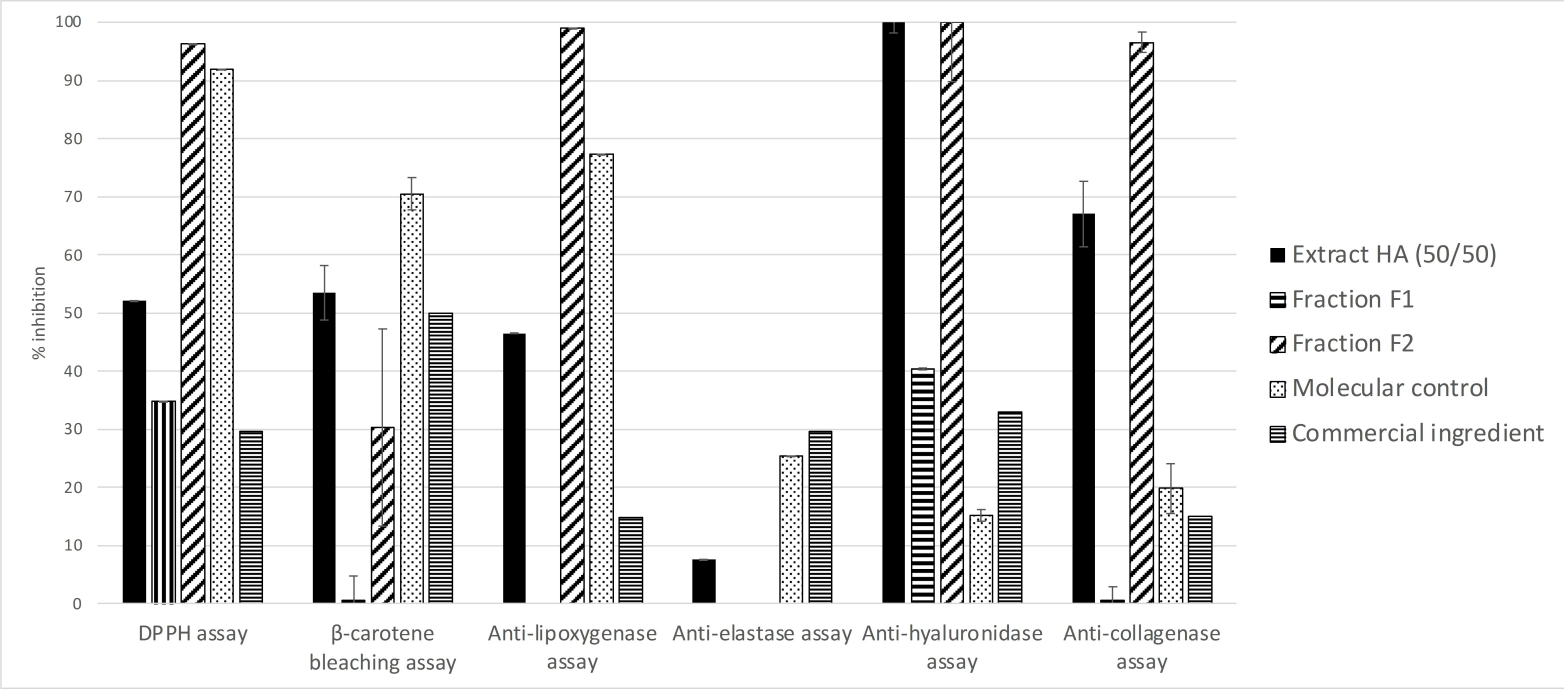
Bioactivities of the hydroalcoholic extract of *O. carpinifolia* twigs and of the fractions F_1_ and F_2_ compared to positive controls. All values are mean ± SEM, done in triplicate.

The two groups of molecules called F_1_ and F_2_ in **Figure** 3, were separated by C_18_ chromatography as described in the experimental section. A yield of 61 % was obtained for Fraction 1 and a yield of 37 % for Fraction 2. The success of this separation was confirmed by HPLC‐DAD/ELSD analysis under same conditions as the hydroalcoholic 50/50 extract. The bioactivities of these fractions were evaluated in the same conditions as those applied to the hydroalcoholic 50/50 extract using the seven bioassays. As presented in **Figure** 4, **F1** presents some antioxidant and anti‐hyaluronidase biological activities but always weaker than the crude extract and any anti‐lipoxygenase and elastase activities. In contrast, F_2_ displays β‐carotene bleaching activity quite similar to the hydroalcoholic 50/50 extract and stronger antioxidant, anti‐lipoxygenase, anti‐collagenase and anti‐hyaluronidase activities. We observe that the anti‐elastase activity was lost in F_1_ and F_2_ independently. The iron reducer power was about 0.62±0.16 mM/LFe(II) for F_1_ and about 17.81±1.81 mM/LFe(II) for F_2_. The latter value is greater than for the hydroalcoholic extract. This led to the conclusion that the hydroalcoholic 50/50 extract's activities are mainly conferred by compounds in F_2._

**Figure 5:**
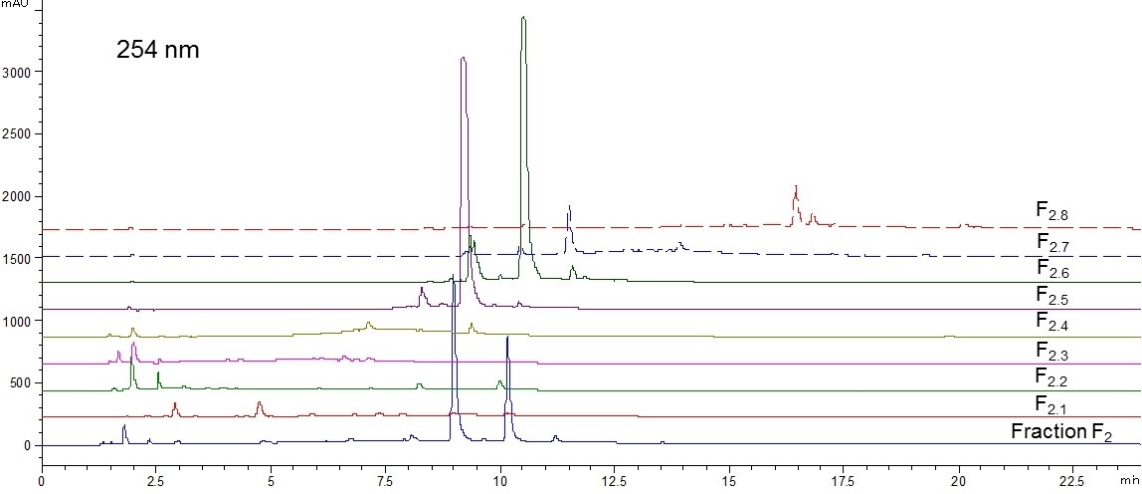
HPLC chromatograms at 254 nm of fraction F_2_ and of the eight corresponding sub‐fractions obtained with gradient 2.

**Figure 6:**
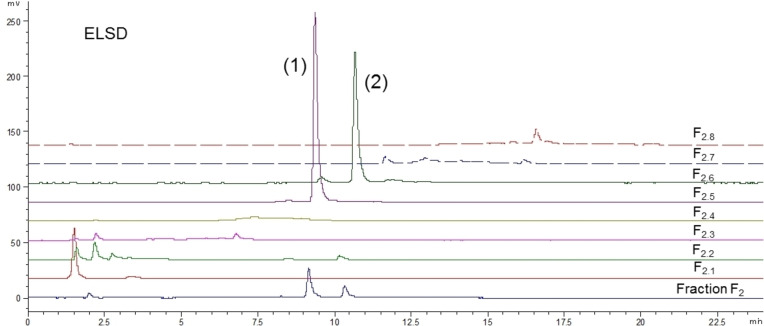
HPLC ELSD chromatograms of fraction F_2_ and of the eight corresponding sub‐fractions obtained with gradient 2.

As mentioned in the introduction, no prior publication has described the phytochemical composition of *O. carpinifolia*. So considering the interesting biological activities observed, bio‐guided fractionation of the active fraction F_2_ was carried out. F_2_ was purified by C18 open column chromatography into eight sub‐fractions numbered F_2.1_ to F_2.8_ and phytochemical compositions of these sub‐fractions were analyzed by HPLC‐DAD/ELSD (**Figure** 5 and **Figure** 6).

These analyses highlighted the relative richness in phenolic compounds with highly diverse polarities, and hence structural diversity. However, those molecules are only present in very low amounts compared to the major compounds (**1**) (t_R_=9.3 min) and (**2**) (t_R_=10.5 min), which were properly separated and concentrated in fractions F_2.5_ and F_2.6_ respectively.

**Figure 7:**
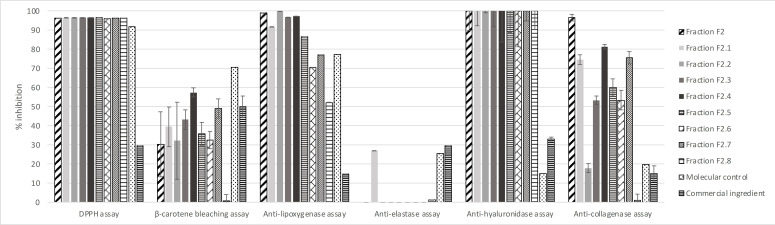
Bioactivities of the eight sub‐fractions compared with positive controls. All values are mean ± SEM, done in triplicate.

Bioactivities of these eight sub‐fractions were evaluated as described for fractions F_1_ and F_2_; results are presented in **Figure** 7.

We observe that all the sub‐fractions possess robust antioxidant and anti‐hyaluronidase biological activities equivalent to the mother fraction F_2_. Sub‐fractions F_2.1_ to F_2.7_ present similar or stronger β‐carotene bleaching activity, only F_2.8_ shows weaker activity. For anti‐lipoxygenase activity, only F_2.2_ presents results similar to F_2_, while other sub‐fractions present results from 97 % for F_2.7_ to 51 % for F_2.8_. All the compounds may be useful for an anti‐pollution cosmetic active, considering the result of 14±0,01 % for the positive control. The anti‐elastase activity of 27±0.04 % inhibition (F_2.2_) is notable because F_2_ displays none. Finally, all sub‐fractions except F_2.2_ and F_2.8_ present potentially useful anti‐collagenase activities, although they are at least 15 % weaker than the initial faction F_2_. Fractions F_2.2_, F_2.3_, F_2.4_, F_2.5_ and F_2.6_ show similar or better iron reducer powers than F_2_ (around 17 mM/LFe(II)) and F_2.3_ has a somewhat higher iron reducer power of 24.89±2.19 mM/LFe(II). Fractions F2.1, F2.7 and F2.8 show lower iron reducer powers of 11.5±1.37, 9.02±1.21 and 0.20±0.06 mM/LFe(II), respectively.

While compounds (**1**) and (**2**) of the hydroalcoholic 50/50 extract are the main components of F_2.5_ and F_2.6_, respectively, it appears they are not responsible for the strong anti‐pollution activities of *O. carpinifolia* on their own (**Figure** 7). The full spectrum therefore seems to have more potential than isolated compounds for developing an active cosmetic ingredient, because of the positive synergy existing between major and minor compounds of *O. carpinifolia*.

However, to further investigate the phytochemistry of *O. carpinifolia* and to assess the bioactivities of the two major pure compounds, isolations of these two molecules were undertaken.

### Isolation and StructuralElucidation of the Two Main Compounds of *O. carpinifolia* Hydroalcoholic Extract

Fractionation of sub‐fractions F_2.5_ and F_2.6_ by semi‐preparative HPLC as described in the experimental section led to the isolation of the two compounds (**1**) and (**2**), purity superior to 80 %, from the hydroalcoholic 50/50 (v/v) extract of *O. carpinifolia*. Firstly, analysis of their UV spectrum suggested that they were flavonoids. Then, these two compounds were analyzed by UHPLC‐HRMS and NMR spectroscopy. For both compounds (**1**) and (**2**), a neutral loss of 146 Da in the MS/MS spectra suggested the loss of a rhamnose moiety. Presence of rhamnose was confirmed by comparison of the ^1^H and ^13^C NMR data with previous studies. NMR data also made it possible to find all the characteristic signals of flavonoid genins for compounds (**1**) and (**2**) with the only difference between them being a signal corresponding to a hydroxy group in place of the hydrogen on ring B of compound (**2**). Therefore, combining HRESIMS and NMR data and comparing the spectroscopic data with previously published works, we identified compound (**1**) as **myricitrin** and compound (**2**) as **quercitrin**.

### *In vitro* Activities of Isolated Compounds

Once isolated and identified as previously described, the two compounds myricitrin (**1**) and quercitrin (**2**) (at 100 μg/mL) were evaluated using the same set of selected bioassays.

These two molecules are widely distributed in the plant kingdom and have already been described in several different families, for example, *Myrica rubra* (Myricaceae),^[31]^ *Eugenia edulis* (Myrtaceae)^[32]^ and *Nymphaea caerulea* (Nymphaeaceae),^[33]^ for myricitrin, and *Kalanchoe pinnata* (Crassulaceae)^[34]^ and *Fagopyrum tataricum* (Polygonaceae),^[35]^ for quercitrin. Both compounds have also been previously studied for their biological activities. Hence, the antioxidant potential of quercitrin has already been described *in vitro*[^36^, ^37^] and on cells,^[38]^ as well as being associated with reparative power against various damage induced by UVB exposure.^[18]^ The anti‐lipid peroxidation potential against the production of TBARS (Thiobarbituric acid reactive substances) of quercitrin has also been demonstrated in mouse brains by Wagner et al. (2006).^[39]^ Reactive substances resulting from lipid peroxidation react with thiobarbituric acid to form TBARS, thus allowing the quantification of oxidative stress. Myricitrin is also widely known for its antioxidant activities.[^37^, ^40^, ^41^, ^42^] According to the literature, other biological activities are attributed to these two compounds, but they are not related to the cosmetic domain.

Myricitrin and quercitrin are polyphenols with significant bioactivity, making them compelling compounds for toxicity and genotoxicity studies.[^42^, ^43^] However, it is essential to conduct more extensive toxicity testing on the complete extract, particularly if it is intended for commercialization.

**Figure 8:**
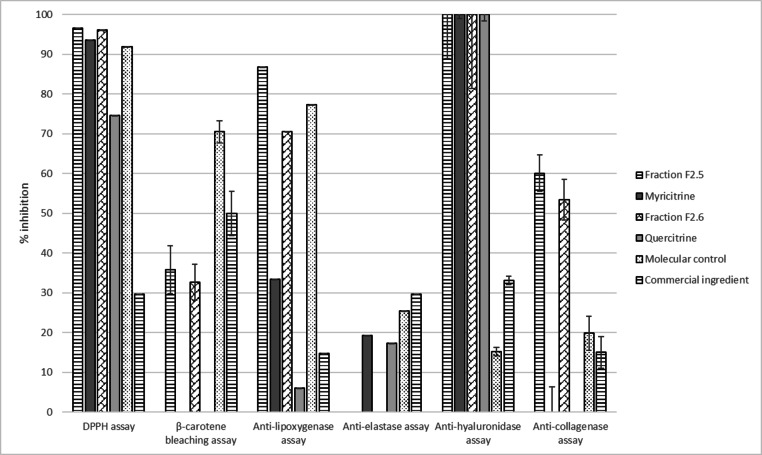
Bioactivities of the two major compounds of the hydroalcoholic 50/50 extract, myricitrin (1) and quercitrin (2), compared to the positive control. All values are mean ± SEM, done in triplicate.

Results presented in **Figure** 8 demonstrate that myricitrin seems to be responsible for the excellent DPPH and anti‐hyaluronidase activities of F_2.5_ but not for the β‐carotene bleaching, anti‐lipoxygenase and anti‐collagenase activities. Quercitrin also shows good antioxidant and anti‐hyaluronidase properties, though less active than F_2.6_, but little activity in all other assays. Myricitrin and quercitrin showed a weak anti‐elastase activity that was completely nonexistent in F_2.5_ and F_2.6_. This may be due to the tested concentration; in the extract, these molecules may be concentrated at less than 1 μg/mL and therefore may not be concentrated enough to exhibit these activities. The iron reducer powers of myricitrin and quercitrin (15.14±1.62 and 17.88±1.70 mM/LFe(II), respectively) are quite similar to each other and to F_2_. These results support our earlier conclusion that myricitrin and quercitrin are not the origin of the good anti‐pollution activities of *O. carpinifolia* on their own. Therefore it will be more useful to use the crude extract or fraction F_2_ to develop an effective anti‐pollution cosmetic active from *O. carpinifolia*.

### Development of Cosmetic Actives Based on *Ostrya carpinifolia* Extracts

Having established the interesting biological anti‐pollution properties of the hydroalcoholic extract of *Ostrya carpinifolia*, we now focus on the development of the cosmetic active, in particular the optimization of the extraction process. The main goal of this optimization is to obtain the most active extract with the best possible yield in the least number of steps and thus the simplest and easiest process applicable on a large scale, i. e. on an industrial scale. It is also important to be aware that, most of time, extracts are not directly incorporable into a cosmetic formulation mainly due to its viscosity after concentration. It must be transformed to facilitate its incorporation in cosmetic formulas by deposition on an adequate support, which can be either liquid or solid.

In the optimization of an extract, yield is the primary consideration when selecting a new solvent. While other factors also play a role, ethanol and hydroalcoholic solvents are historically favored by cosmetic companies. A yield above 8 % is considered good, which supports the continued use of these solvents. However, if the yield is below 8 %, as is the case here (less than 6 %), this is seen as disappointing, prompting the exploration of alternative solvents.

Despite this, phytochemical analysis is easier to perform on extracts obtained with ethanol and hydroalcoholic solvents, allowing for the identification of molecules like polyphenols.

To meet the demands of the cosmetics industry, it has become increasingly common over the past several years to use solvents that are not removed during processing. These solvents can thus serve as both carriers and active or excipient components within a formulation. Various solvents, such as glycol mixtures or hydro‐glycerinated blends, are generally employed for this purpose.

In particular, propylene glycol has demonstrated the best bioactivities, making it a preferred solvent, especially as it is well‐regarded by cosmetic companies.

Solid cosmetic ingredients consist of an optimized extract which, once concentrated, is deposited on a powder‐type support such as maltodextrin for example. Liquid cosmetic ingredients can be made from the same optimized extract but this time resolubilized in a solvent that can be easily used by formulators or obtained by direct maceration in an appropriate solvent. Development of both solid and liquid cosmetics is a real advantage to meet the demands that formulators might have, especially in a context where solid cosmetic products (shampoos, care products, etc.) are becoming more and more popular.^[44]^

### Solid Cosmetic Ingredient

It has been shown that the molecules responsible for the biological activities of the hydroalcoholic extract have intermediate polarity. To obtain a more active extract, one can adjust the ethanol/water ratio of the extraction solvent. We compared EtOH, hydroalcoholic 80/20 and hydroalcoholic 50/50 extracts (**Table** 2). Extraction yields obtained using hydroalcoholic 80/20 and 50/50 mixtures did not differ substantially and were considerably higher than that obtained with ethanol alone.

**Table 2:** Extraction yields obtained using ethanol alone and hydroalcoholic 80/20 and 50/50 mixtures

Solvent	EtOH	HA (80/20)	HA (50/50)
Yield (%)	2.53±0.65	5.04±0.19	6.04±0.24

**Figure 9:**
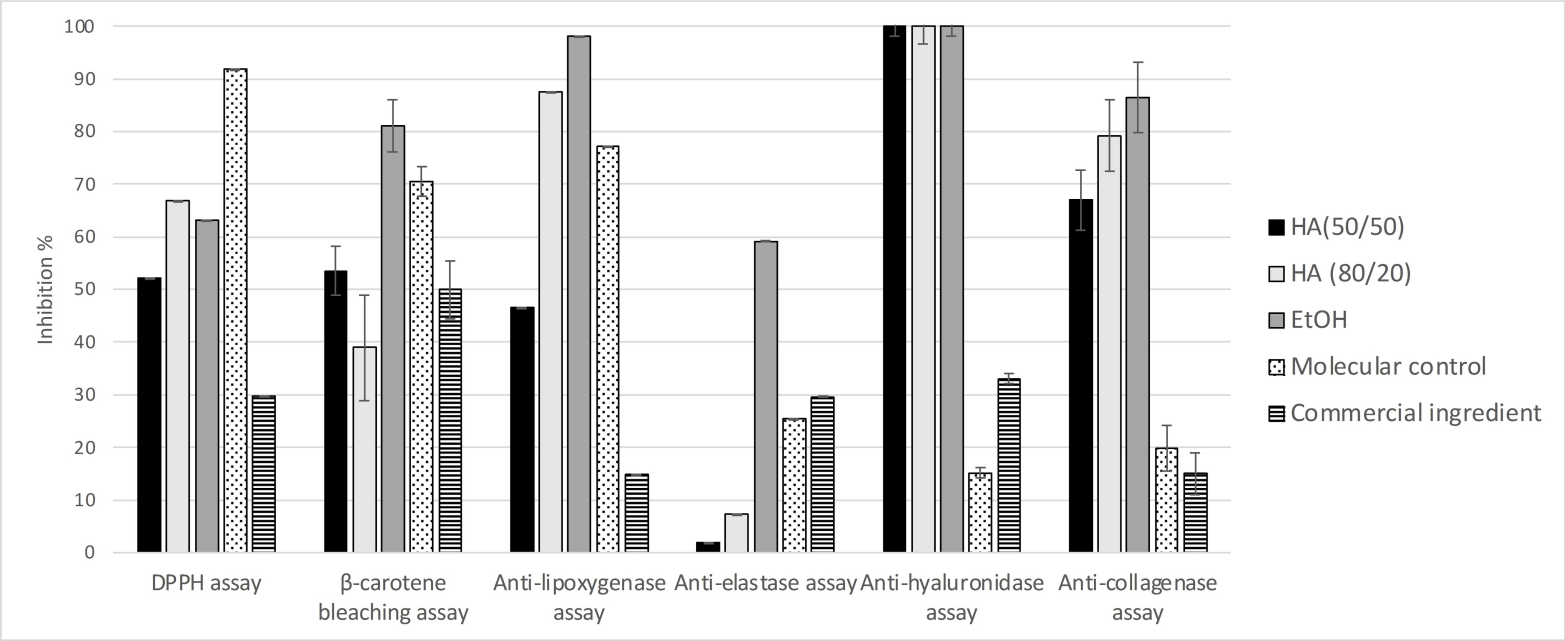
Bioactivities of the EtOH, hydroalcoholic 80/20 (HA (80/20)) and hydroalcoholic 50/50 (HA (50/50)) of *O. carpinifolia* compared to positive controls. All values are mean ± SEM, done in triplicate.

The bioactivities of these three extracts were then evaluated (**Figure** 9). The three extracts display quite similar anti‐hyaluronidase activities. However, we observe that the anti‐lipoxygenase activity of HA (50/50) extract is two times weaker than those of the two other extracts. Furthermore, the EtOH extract displays a strong elastase activity not previously observed in any of the *O. carpinifolia* extracts and fractions we studied. The EtOH extract shows a better β‐carotene bleaching activity than the other hydroalcoholic extracts. Also, results of antioxidant and anti‐collagenase activities are twice as strong for the HA (80/20) and EtOH extracts as for the HA (50/50) extract. The iron reducer powers are quite similar between all three extracts. The HA (80/20) extract shows the best iron reducer power, with 8,20±1.18 mM/LFe(II).

According to these results, the development of a cosmetic active should preferentially be undertaken using an *O. carpinifolia* extract obtained with a hydroalcoholic 80/20 mixture. The extraction yield obtained with ethanol 100 % is too low to be viable for an industrial development. Moreover, a lower quantity of water in the extraction solvent makes it possible to reduce the energy‐consumption and the pressure needed during the concentration step of the extraction process.

The dry extract we have developed requires additional investigation, particularly concerning its formulation with a carrier suitable for cosmetic applications. To expand the options for formulators and align with the trend towards solid cosmetics, exploring the incorporation of this active ingredient into solid forms using maltodextrin supports holds promise, and will be the scope of another study. This may include developing an atomization process. The process of formulating an extract onto a solid support presents unique challenges, including ensuring proper dispersion and stability of the active ingredient within the matrix. Additionally, considerations such as maintaining the efficacy and bioavailability of the extract throughout the formulation process must be addressed to achieve the desired cosmetic outcomes.

### Liquid Cosmetic Ingredient

**Figure 10:**
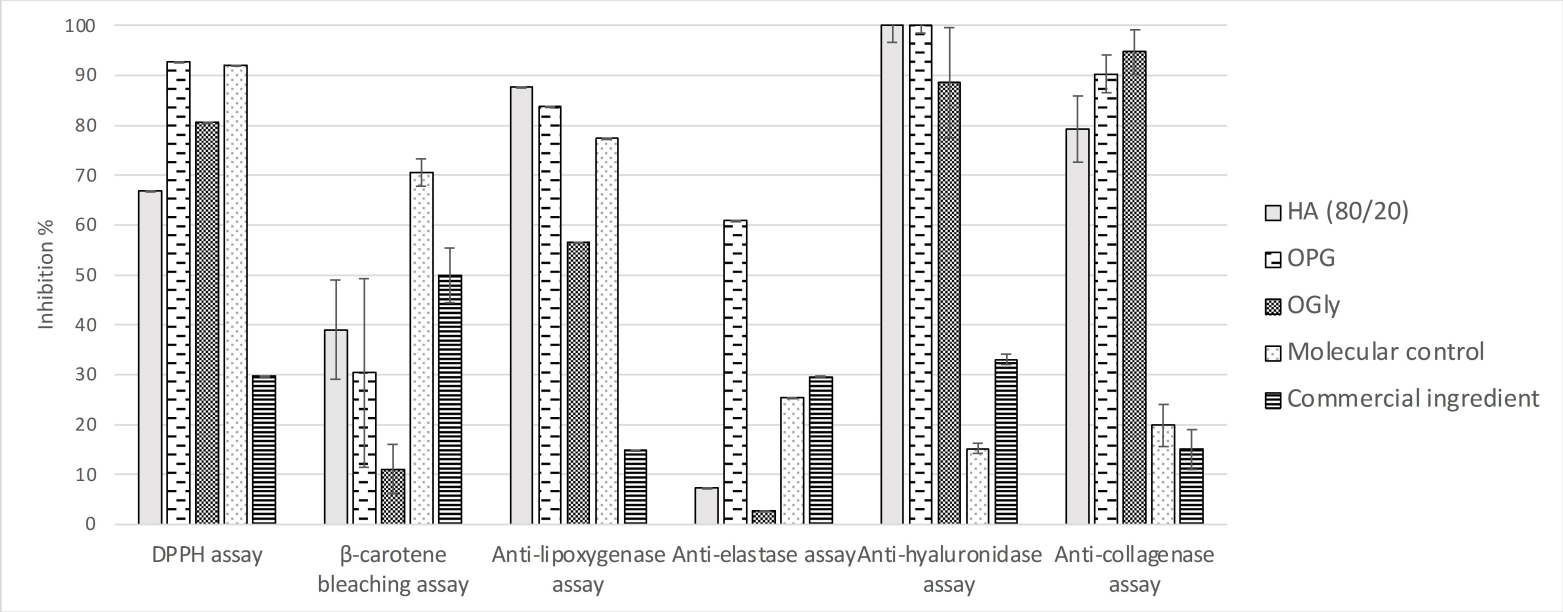
Bioactivities of the propylene glycol (OPG) and glycerin (OGly) extracts of O. carpinifolia compared to hydroalcoholic 80/20 extract (HA (8/2)) and positive controls. All values are mean ± SEM, done in triplicate.

The most common form for cosmetic ingredients remains the liquid form due to the inherent ease of incorporating such forms in formulations. Glycerin and propylene glycol are the most widely used liquid supports, and serve as texturizers in many personal care formulations including facial cream, lotion, mist, cleanser, etc..[^45^, ^46^] These solvents were thus used to obtain extracts OPG and OGly through direct maceration of ground *O. carpinifolia* twigs in propylene glycol and glycerin, conducted at ambient temperature with constant agitation, to assess their bioactivities *in vitro* (**Figure** 10). Both solvents seem to be quite promising to use in view of the activities demonstrated for both these extracts. While both extracts display quasi‐equivalent anti‐hyaluronidase and β‐carotene bleaching activities (100.00±1.86 % initial value) compared to that of the HA (80/20) extract, their antioxidant and anti‐collagenase activities show more potential. Though a little lower than that of the HA (80/20) extract, their anti‐lipoxygenase activities are still promising. Moreover, we note that the OPG anti‐elastase activity is over 60 %, considerably higher than that of the commercial ingredient tested in the same conditions (**Figure** 9). The iron reducer power has been tested too but it shows low activity, around 0.20±0.05 mM/LFe(II) for glycerin and propylene glycol extracts (in comparison with commercial extracts with 0.42±0.005 mM/LFe(II)).

**Figure 11:**
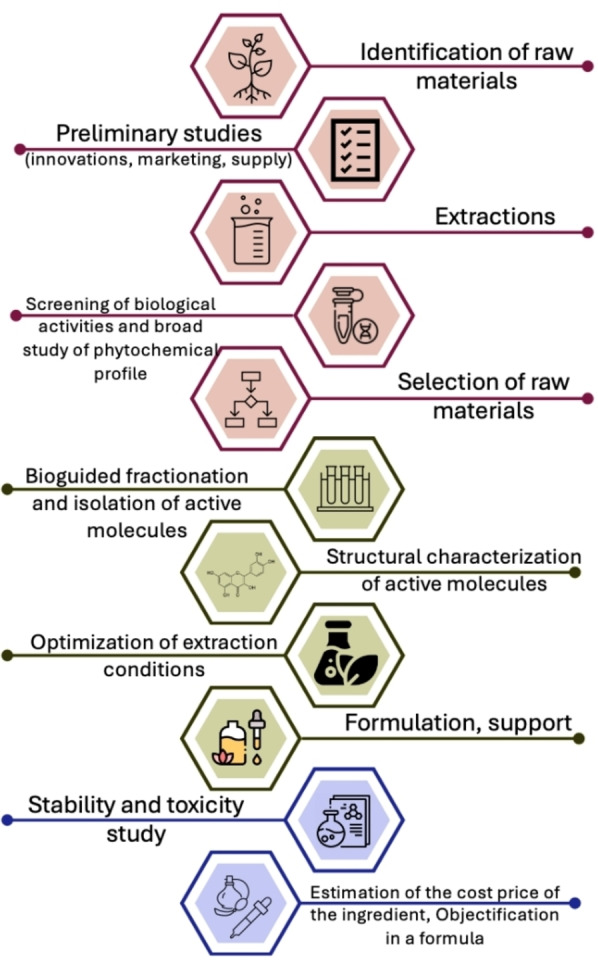
Steps in the development of a cosmetic product.

The steps involved in developing an active ingredient are numerous (**Figure** 11); natural extracts are typically not directly added to cosmetics. Before formulation, studies on solubility, discoloration, deodorization, and stability must be conducted.

The development of an ingredient continues to be a lengthy process due to the need for further optimization of extraction conditions, testing for toxicity, and conducting more comprehensive *in vitro* or *in vivo* studies to confirm the demonstrated activities. These essential steps are crucial for ensuring the safety, efficacy, and regulatory compliance of the ingredient before it can be incorporated into cosmetic formulations.

## Conclusions

This study comprehensively evaluated the potential of *Ostrya carpinifolia* L. as an anti‐aging and anti‐pollution cosmetic active ingredient through a series of seven bioassays, including Hydrogen Atom Transfer (HAT), Single Electron Transfer (SET), and Mixed HAT/SET system assays. A 50/50 hydroalcoholic extract demonstrated promising biological activities relevant to the cosmetic industry, prompting further investigation using a bio‐guided fractionation approach. The biological activities were largely driven by the polyphenolic fraction, with two major compounds isolated and identified as myricitrin (1) and quercitrin (2). This is the first report of these compounds being extracted, purified, and evaluated within the *Ostrya* genus. Notably, the study revealed that the observed biological effects were not solely due to these two compounds, but rather to a synergistic interaction between various polyphenolic metabolites within the 50/50 hydroalcoholic extract.

The significance of these findings lies in the anti‐pollution potential of *O. carpinifolia*, as its ability to combat oxidative stress and skin damage induced by pollutants was confirmed. This positions the species as a promising candidate for industrial development in cosmetic formulations aimed at mitigating pollution‐related skin aging.

To advance toward industrial application, various extraction solvents were tested to optimize both yield and biological activity. The 80/20 hydroalcoholic extract outperformed the 50/50 extract in terms of both activity and yield, while the ethanolic extract exhibited stronger biological results but with insufficient yield for large‐scale production. Glycerin and propylene glycol extracts also showed excellent potential, particularly for liquid active ingredients, offering flexibility for future cosmetic formulations. Given the growing interest in solid cosmetics, exploring solid forms of the extract using maltodextrin supports and atomization processes would be a valuable next step.

For industrial development, the next phase involves scaling up production through pilot batches to validate the optimized extraction protocols and ensure the consistency of biological activities. Stability and cytotoxicity studies will follow, laying the groundwork for the commercialization of *O. carpinifolia* as a versatile anti‐aging and anti‐pollution cosmetic active ingredient.

As a result, this study lays a solid foundation for further development and commercial use of *O. carpinifolia* in innovative cosmetic formulations aimed at protecting skin from environmental damage.

## Experimental Section

### Materials and Methods

#### Plant Material

Twigs of *O. carpinifolia* L. were collected and botanically identified by François Boileau, botanist, from the Biophyto association, in the Valon de la Cagne terraces, Saint‐Jeannet, France, at an altitude of 200 m, in June 2018. A voucher specimen of *O. carpinifolia* was deposited in the herbarium collection of the Natural History Museum of Nice, France, under the reference number NICE−D‐5321.

The chemicals and solvents were purchased from various companies, including Sigma‐Aldrich Merck, VWR Prolabo, and Dutscher.

### Extraction and Fractionations

#### Extraction of the Twigs of *Ostrya carpinifolia*

The air‐dried twigs of *O. carpinifolia* were first ground to obtain a homogenous powder. For the first part of this study, which involved bio‐guided phytochemical analysis, 203.75 g of powder of *O. carpinifolia* was extracted by 2 successive macerations using an hydroalcoholic solvent (EtOH/H_2_O 50/50 v/v, plant material/solvent ratio: 1/5) for 2 h at room temperature under agitation (500 rpm). After filtration over filter paper 8–12 μm, the residue was concentrated under reduced pressure to give 29.45 g of hydroalcoholic 50/50 extract, a yield of 14.45 %.

For the second part of the study, to be consistent with any future development and industrial implementation of a cosmetic active ingredient, powder of *O. carpinifolia* was extracted only once with the hydroalcoholic (EtOH/H_2_O; 80/20 and 50/50; v/v) solvents and ethanol alone, using the same plant material/solvent ratio 1/5, for 2 h at room temperature under agitation (500 rpm). Extractions were carried out in triplicate for this part.

Also in the ingredient development phase, when other solvents with higher viscosity and weaker polarity like glycerine and propylene glycol solvents were used, a higher mass ratio plant/solvent of 1/10 was chosen, and we carried out the maceration for 8 h at room temperature under agitation (500 rpm).

### Fractionation of the Crude Extract

The crude extract of the twigs of *O. carpinifolia* was fractionated over C18‐silica gel (30 g) using distilled water (200 mL) and methanol (200 mL) to obtain two fractions. F1 was collected using MeOH/H_2_O 0/100 (v/v) and F2 was collected using MeOH/H_2_O 80/20 (v/v) and MeOH/H_2_O 100/0 (v/v).

The fractionation was repeated ten times on the same column; the silica gel was rebalanced with 150 mL of distilled water before each deposition of 1 g of crude extract. The resulting fractions F1 comprising the higher polar compounds, and F2, all other compounds, were respectively pooled together to obtain a total of 6.1 g of fraction F1 (recovery yield: 61 %) and of 3.7 g of fraction F2 (recovery yield: 37 %).

#### Solid Phase Extraction (SPE)

In order to study phytochemistry by HPLC of extracts obtained with high‐viscosity solvents such as glycerine and propylene glycol, it is necessary to concentrate the fraction using solid phase extraction. SPE cartridges (Supelco, Discovery® DSC‐18, 500 mg/6 mL) were fixed on a vacuum manifold. The solid phase was first conditioned with 10 mL methanol (MeOH) and then, equilibrated with 10 mL of distillate water. The extract was diluted in water (extract/water 1/3 v/v), to permit the elution blocked by high viscosity of glycerine and propylene glycol, and was loaded on the SPE cartridge. After sample deposition, elution was performed using 10 mL MeOH, 10 mL of MeOH/dichloromethane (1/1, v/v) followed by 10 mL dichloromethane. The resulting fractions were pooled and concentrated under reduced pressure to obtain the extract.

### Fractionation of Fraction F_2_

Fraction F_2_ was sub‐fractioned over the same C18‐silica gel using 200 mL of 8 different hydroalcoholic mixtures. This operation was repeated three times to fractionate a total of 2.78 g of fraction F2. Finally, 470 mg of sub‐fraction F_2.1_ was collected using MeOH/H_2_O 0/100 (v/v), 240.5 mg of F_2.2_ using MeOH/H_2_O 10/90 (v/v), 397.4 mg of F_2.3_ using MeOH/H2O 20/80 (v/v), 390.5 mg of F_2.4_ using MeOH/H2O 30/70 (v/v), 505 mg of F_2.5_ using MeOH/H_2_O 40/60 (v/v), 333.4 mg of F_2.6_ using MeOH/H_2_O 50/50 (v/v), 104 mg of F_2.7_ using MeOH/H_2_O 60/40 (v/v) and 36.7 mf of F_2.8_ using MeOH/H_2_O 70/30 (v/v).

Compounds (**1**) and (**2**) were purified by semi‐preparative HPLC (purity superior to 80 %).

Myricitrin (**1**): colorless oil, HRESIMS *m/z* 465.1012 [M+H]^+^ Δppm=−1.5 (calculated for C_21_H_21_O_12_^+^, 465.1038); ^1^H and ^13^C NMR data (MeOD, 500 MHz) : δ 6.20 (1H, d (2.2); H‐6), δ 6.37 (1H, d (2.2); H‐8), δ 6.97 (1H, s; H‐2’), δ 6.22 (1H, d (2.2); H‐6’), δ 5.34 (1H, br s; H‐1′′), δ 4.24 (1H, dd (1.8 ; 3.6); H‐2′′), δ 3.81 (1H, dd (3.6 ; 9.6); H‐3′′), δ 3.37 (1H, m; H‐4′′), δ 3.53 (1H, m; H‐5′′), and δ 0.98 (3H, d (6.3); H6′′). The molecule was identified based on data found in the literature.^[47]^

Quercitrin (**2**): colorless oil, HRESIMS *m/z* 449.1061 [M+H]^+^ Δppm=−3.9 (calculated for C_21_H_21_O_11_^+^, 449.1088); ^1^H and ^13^C NMR data (MeOD, 500 MHz) : δ 6.20 (1H, d (2.2); H‐6), δ 6.37 (1H, d (2.2); H‐8), δ 7.33 (1H, d (2.3); H‐2’), δ 6.90 (1H, d (8.4); H‐5’), δ 7.30 (1H, dd (2.2 ; 8.3); H‐6’), δ 5.34 (1H, d (1.8); H‐1′′), δ 4.21 (1H, dd (1.9 ; 3.6); H‐2′′), δ 3.74 (1H, dd (3.6 ; 9.5); H‐3′′), δ 3.33 (1H, m; H‐4′′), δ 3.42 (1H, m; H‐5′′), and δ 0.93 (3H, d (6.4); H6′′). The molecule was identified based on data found in the literature.^[48]^

### Analysis

HPLC‐DAD/ELSD analyses were performed using an HPLC Agilent 1200 system (Courtaboeuf, Ile‐de‐France, France) equipped with a Luna® column C18 100 Å (150×4.6 mm, 5 μm) (Phenomenex, Le Pecq, Ile‐de‐France, France), a Diode Array Detector system and an Evaporative Light Scattering Detector. Several elution gradients were used in this study; details are given below.

Gradient 1 consisted of A: water and B: acetonitrile, both acidified with 0.1 % formic acid, and C: propan‐2‐ol; 0–4 min, 2 % B; 4–15 min, 2–98 % B; 15–20 min, 98 % B; 20–25 min, 98–2 % B and 2–98 % C, 25–30 min, 2 % B and 98 % C, 30–32 min, 0–98 % A, 2 % B and 98–0 % C (flow rate: 1 mL/min).

Gradient 2 consisted of A: water and B: acetonitrile both acidified with 0.1 % formic acid: 0–1 min, 10 % B; 1–20 min, 10–60 % B; 20–21 min, 60 % B; 21–23 min, 60–10 % B, 23–24 min, 10 % B (flow rate: 1 mL/min).

#### Semi‐Preparative HPLC

Semi‐preparative HPLC was performed on the same system as the HPLC‐DAD/ELSD analysis but equipped with a Luna® column C18 100 Å (10×250 mm, 5 μ; Phenomenex, Le Pecq, Ile‐de‐France, France).

Gradient 3 consisted of A: water and B: acetonitrile, both acidified with 0.1 % formic acid: 0–1 min, 30 % B; 1–20 min, 30–40 % B; 20–35 min, 40–30 % B; 35–36 min, 30 % B (flow rate: 3 mL/min).

#### UHPLC‐MS Analysis

UHPLC was performed on a Vanquish system. High resolution (HR)‐ESI‐MS spectra were acquired on a Q Extractive Focus mass spectrometer equipped with an Electrospray Ionization Source (ESI) and a Sphinx® column C18‐phenyl X Å (150×4.6 mm, 3 μm). Ionization was carried out in both negative and positive modes. The mass spectrometer scanned a range of *m/z* between 80 and 1200 with a resolution fixed at 35.000. The oven temperature was set at 25 °C and flow rate fixed at 0.3 mL/min.

Gradient 4 consisted of A: water and B: acetonitrile, both acidified with 0.05 % formic acid: 0–5 min, 5 % B; 5–40 min, 5–45 % B; 40–50 min, 45–100 % B; 50–55 min, 100 % B; 55–60 min, 100–5 % B.

#### Nuclear Magnetic Resonance (NMR) Analysis

NMR spectra were acquired on both 400 MHz and 500 MHz Bruker® Avance NMR spectrometers (Bruker, Fällanden, Switzerland) using TMS as reference. Samples were analyzed in deuterated methanol at 10 mg/mL concentration.

### Bioassays

#### Materials

The bioassays were performed in untreated 96‐well plates from Thermo Nunc (Villebon‐sur‐Yvette, Ile‐de‐France, France), except for the lipoxygenase bioassay which was performed in UV‐transparent 96‐well plates from Costar, Sigma‐Aldrich (Saint‐Quentin Fallavier, Auvergne‐Rhône‐Alpes, France). During agitation and incubation, the 96‐well plates were sealed with adhesive films (Greiner Bio‐One, Courtaboeuf, Ile‐de‐France, France).

#### Sample Preparation

All the samples were prepared at a concentration of 3.433 mg/mL in dimethyl sulfoxide (DMSO, Sigma Aldrich) to obtain a final concentration of 100 μg/mL in the well after deposit of all reagents. DMSO was tested alone in each plate and constitutes the negative control. A commercial extract currently used in the cosmetic sector was also prepared at a concentration of 3.433 mg/mL in DMSO and was added to each plate (**Table** 3). All the samples were tested in triplicate.

**Table 3:** Positive control used in the *in vitro* bioassays.

Bioassay	Positive control.	Commercial extract (3,433 mg/mL in DMSO)
DPPH radical scavenging assay	Resveratrol (3.433 mg/mL in DMSO)	*Rosmarinus officinalis* L. commercial extract
FRAP assay	Fe^II^ (1 mg/mL to 0.025 mg/mL in water)	*Rubus idaeus* L. commercial extract
β‐carotene bleaching assay	BHT (70 mg/mL in EtOH)	*Camellia sinensis* commercial extract
Lipoxygenase assay	Resveratrol (3.433 mg/mL in DMSO)	*Arnica montana* L. commercial extract
Elastase assay	Quercetin (2.59 mg/mL in DMSO)	*Rubus idaeus* L. commercial extract
Hyaluronidase assay	Resveratrol (3.433 mg/mL in DMSO)	*Rubus idaeus* L. commercial extract
Collagenase assay	Tannic acid (2.93 mg/mL in DMSO)	*Rubus idaeus* L. commercial extract

All molecular standards, including resveratrol, iron, BHT, quercetin, and tannic acid, were purchased from Sigma Aldrich, while the cosmetic controls were sourced from Crodarom and Clearstem.

### Instrumentation

Deposition of the samples in the plate was performed by an automated pipetting system Eppendorf epMotion® 5070 (Eppendorf France SAS, Montesson, Ile‐de‐France, France). Optical density (OD) measurements were performed using a Spectramax Plus 384 microplate reader (Molecular Devices, Wokingham, Berkshire, UK) controlled by the SoftMaxPro software (Molecular Devices, Wokingham, Berkshire, UK). Calculations of inhibition percentages were carried out with Prism software (GraphPad Software, La Jolla, CA, USA) and, except for the FRAP assay, the result was expressed as the concentration of antioxidants having a ferric reducing ability equivalent to that of 1 mmol/L FeSO_4_. The results of the other assays expressed as inhibition percentages (I%) were calculated as follows:

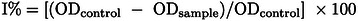




(for the DPPH, lipoxygenase and elastase assays),

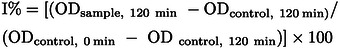




(for the β‐carotene bleaching), or:

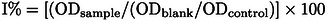




(for the hyaluronidase and collagenase assays),

with OD_control_ corresponding to the optical density of DMSO alone or EtOH alone for the β‐carotene bleaching, OD_sample_ to the optical density of the sample evaluated and OD_blank_ to the optical density before the reagent addition.

For assays using the first formula, i. e., the one not taking account of the OD_blank_ value, a correction considering the OD the sample before addition of the substrate was automatically undertaken on all samples to take the influence of the sample's color into consideration.

### DPPH Radical Scavenging Assay

This assay was inspired by the protocol described by Popovici *et al.*.^[49]^ A volume of 150 μL of ethanol/acetate 50/50 (v/v) buffer was added to each well, along with 7.5 μL of either the sample or the control. An OD reading (OD_blank_) was performed at 517 nm before introducing 100 μL of an ethanolic solution of 1,1‐diphenyl‐2‐picrylhydrazyl radical (DPPH; 386.25 μM). Following that, the plate was then sealed and incubated for 30 min in the dark before final OD reading at 517 nm (OD_sample_).

### FRAP Assay

This was inspired by the protocol described by Benzie and Strain.^[18]^ An amount of 150 μL of acetate buffer pH=3.6 was deposited in each well together with 7.5 μL of the sample or the control. An OD reading (OD_blank_) was performed at 593 nm before deposition of 100 μL of a mixture of FRAP solution in acetate buffer, 2,4,6‐tripyridyl‐s‐triazine 10 mM in HCl 40 mM and FeCl_3_.6H_2_O 20 mM in H_2_O prepared in volumetric proportions 10/1/1. The plate was then sealed and incubated for 4 min in the dark before a final OD reading at 593 nm. A calibration curve was prepared with Fe(II) solutions of different concentrations ranging from 0.04 and 3.6 mM and the results of the samples are calculated by projection on the calibration curve.

### β‐carotene Bleaching Assay

Inspired by the protocol described by Koleva *et al*.,^[50]^ an amount of 43 μL of the sample or control, 70 μg/mL in EtOH, was deposited. A β‐carotene solution was prepared as follows: A solution of 0.5 mg of β‐carotene in 1 mL chloroform was mixed with 25 μL of linoleic acid and 200 μL of Tween 40 while protected from light. Chloroform was then evaporated under reduced pressure before the addition of 100 mL of distilled water. Then, 214.6 μL of this solution was added to each well and the OD_0 min_ reading was performed at 492 nm. The plate was incubated at 55 °C for 120 min and a final OD reading was then carried out.

### Anti‐Lipoxygenase Assay

This assay was inspired by the protocol described by Kumaraswamy and Satish.^[51]^ An amount of 150 μL of a soybean lipoxygenase (686.66 U/mL) in phosphate buffer solution was deposited in each well with 7.5 μL of the sample or control. The plate was then sealed to be incubated under agitation in the dark for 10 min before deposition of 100 μL of a solution of linoleic acid (5 μL in 20 mL phosphate buffer). Then, the OD_blank_ reading was performed at 235 nm after 2 min agitation. The final OD reading was performed after 50 min of incubation in the dark.

### Anti‐Elastase Assay

This was inspired by the protocol described by Thring *et al.*.^[52]^ An amount of 150 μL of a solution of porcine pancreatic elastase (0.171 U/mL) in Tris buffer was deposited in each well with 7.5 μL of the sample or control. A first incubation was performed for 20 min before the OD_blank_ reading at 410 nm. Then, 100 μL of a solution of N‐succinyl‐Ala‐Ala‐Ala‐p‐nitroanilide (2.06 mM) in Tris buffer was added. The final OD reading was performed after 40 min of incubation in the dark.

### Anti‐Hyaluronidase Assay

This was inspired by the protocol described by Oueslati *et al.*.^[53]^ An amount of 150 μL of a solution of hyaluronidase (13.3 U/mL) in hyaluronidase buffer was deposited in each well with 7.5 μL of the sample or control. The plate was then sealed to be incubated for 20 min at 37 °C before an OD_blank_ reading at 405 nm. Then, 100 μL of a solution of hyaluronic acid at 150 μg/mL in pH 5.35 buffer (KH₂PO₄ 300 mM) was added to each well before incubation at 37 °C for 30 min. Finally, 50 μL of cetyltrimethylammonium bromide (40 nM in a 2 % NaOH solution) was added and the plate was incubated for 2–10 min before the final OD reading.

### Anti‐Collagenase Assay

This was inspired by the protocol described by Thring *et al.*.^[52]^ An amount of 150 μL of a solution of collagenase (53 U/mL) in tricine buffer (tricine 50 mM, NaCl 400 mM and CaCl_2_ 10 mM, pH 7.5) was deposited in each well with 7.5 μL of the sample or control. The plate was incubated in the dark for 15 min before the OD_blank_ reading at 345 nm. Then, 100 μL of a solution of 2‐furanacryloyl‐l‐leucylglycyl‐l‐propyl‐l‐alanine (5.15 mM) in tricine buffer was added. The final OD reading was performed after 30 min of incubation in the dark.

## Funding Information

This work was supported by the French government through the France 2030 investment plan managed by the National Research Agency (ANR), as part of the Initiative of Excellence Université Côte d'Azur under reference number ANR‐15‐IDEX‐01.

## Conflict of Interests

Declarations of interest: none.

1

## Data Availability

The data that support the findings of this study are available from the corresponding author upon reasonable request.
